# Catalog of Lung Cancer Gene Mutations Among Chinese Patients

**DOI:** 10.3389/fonc.2020.01251

**Published:** 2020-08-04

**Authors:** Xinying Xue, Idorenyin Asuquo, Lei Hong, Jie Gao, Zhouhuan Dong, Li Pang, Tianjiao Jiang, Mingming Meng, Jingbo Fan, Jiaxin Wen, Hui Deng, Xuelei Zang, Xidong Ma, Rui Guo, Chong Qin, Yao Meng, Heji Ma, Jun Han, Haijiao Wang, Zhiqiang Xue, Dahai Zhao, Dongliang Lin, Lei Pan

**Affiliations:** ^1^Department of Respiratory and Critical Care, Beijing Shijitan Hospital, Capital Medical University, Beijing, China; ^2^Department of Radiology, Jinzhou Medical University, Jinzhou, China; ^3^Internal Medicine Department, Xuhui Changqiao Community Health Care Centre, Shanghai, China; ^4^Department of Pathology, Chinese PLA General Hospital, Beijing, China; ^5^Department of Radiology, Affiliated Hospital of Qingdao University, Qingdao, China; ^6^Department of Gastroenterology, Beijing Shijitan Hospital, Capital Medical University, Beijing, China; ^7^Department of Respiratory and Critical Care, Second Affiliated Hospital of Anhui Medical University, Anhui, China; ^8^Department of Chest Surgery, Chinese PLA General Hospital, Beijing, China; ^9^Department of Laboratory, Chinese PLA General Hospital, Beijing, China; ^10^Department of Respiratory and Critical Care, Affiliated Hospital of Weifang Medical University, Shandong, China; ^11^Department of Chest Surgery, Beijing Shijitan Hospital, Capital Medical University, Beijing, China; ^12^Department of Radiology, Third Affiliated Hospital of Chongqing Medical University, Chongqing, China; ^13^Department of Pathology, Affiliated Hospital of Qingdao University, Qingdao, China

**Keywords:** lung cancer, China, gene mutation, aging, tobacco smoking, pathology

## Abstract

**Background:** Detailed catalog of lung cancer-associated gene mutations provides valuable information for lung cancer diagnosis and treatment. In China, there has never been a wide-ranging study cataloging lung cancer-associated gene mutations. This study aims to reveal a comprehensive catalog of lung cancer gene mutations in china, focusing on EGFR, ALK, KRAS, HER2, PIK3CA, MET, BRAF, HRAS, and CTNNB1 as major targets. Additionally, we also aim to correlate smoking history, gender, and age distribution and pathological types with various types of gene mutations.

**Patients and Methods:** A retrospective data acquisition was conducted spanning 6 years (2013–2018) among all patients who underwent lung cancer surgeries not bronchial or percutaneous lung biopsy at three major tertiary hospitals. Finally, we identified 1,729 patients who matched our inclusion criteria.

**Results:** 1081 patients (62.49%) harbored EGFR mutation. ALK (*n* = 42, 2.43%), KRAS (*n* = 201, 11.62%), CTNNB1 (*n* = 28, 1.62%), BRAF (*n* = 31, 1.79%), PIK3CA (*n* = 51, 2.95%), MET (*n* = 14, 0.81%), HER2 (*n* = 47, 2.72%), HRAS (*n* = 3, 0.17%), and other genes(*n* = 232, 13.4%). Females expressed 55.38% vs. males 44.62% mutations. Among subjects with known smoking histories, 32.82% smokers, 67.15% non-smokers were observed. Generally, 51.80% patients were above 60 years vs. 48.20% in younger patients. Pathological types found includes LUADs 71.11%, SQCCs 1.68%, ASC 0.75%, LCC 0.58%, SCC 0.35%, ACC 0.17%, and SC 0.06%, unclear 25.19%.

**Conclusion:** We offer a detailed catalog of the distribution of lung cancer mutations. Showing how gender, smoking history, age, and pathological types are significantly related to the prevalence of lung cancer in China.

## Introduction

Lung cancer is the most common cancer and the leading cause of cancer-related mortality around the world despite extensive concerted study. In China, nearly 3,804,000 (2,114,000 men, 1,690,000 women) lung cancer cases were diagnosed in 2014, which is the equivalent of more than 10,422 cases diagnosed each day (1). High prevalence of driver gene mutations and fusions in EGFR, ALK, RET, ROS1, and KRAS in lung LUAD patients, have been observed in China (2, 3). Especially, point mutations L858R and E746_A750del comprised nearly 90% of all EGFR mutations in NSCLC (4). Notably, non-smoker East Asian women are more likely to develop LUAD and exhibit a higher incidence of EGFR mutation and a lower KRAS mutation frequency (5). Smoking is the leading cause of lung cancer, as <20% of smokers develop this deadly disease in their lifetime but non-smokers with increased risk of lung cancer usually have a family history of cancer. More women suffer from lung cancer. In comparison to the male patients they are younger and more likely never-smokers (6). Age is associated with cancer development due to biologic factors that include DNA damage over time and shortening telomeres (7). Accordingly, the median age of lung cancer diagnosis is 70 years for both men and women. Approximately 53% of cases occur in individuals 55 to 74 years old and 37% occur over 75 years old (8).

In this study we attempted to reveal a detailed catalog of gene mutations in cancer patients within China, detailing EGFR, ALK, KRAS, HER2, PIK3CA, MET, BRAF, HRAS, CTNNB1, and other genes concerning its relationship with gender, age, smoking history, and pathological presentations.

## Methods

A retrospective data acquisition was conducted spanning 6 years (2013–2018) among all patients who attended lung cancer out-patient consultations and underwent lung cancer-related surgeries at three major tertiary hospitals. The data were collected from hospital medical records which comprised clinical medical history, radiology reports, pathology reports, and for some patients whose information was incomplete or incoherent, follow up phone calls were made to ascertain or verify them.

### Statistics

Statistical analyses such as *p-value* calculations were conducted using regression analysis by finding the *R*^2^ values. The *p*-value for each independent variable was used to test the null hypothesis that the variable has no correlation with the dependent variable. An alpha of 0.05 is used as the cutoff for significance. If the *p* < 0.05, we reject the null hypothesis that there's no difference between the means and conclude that a significant difference does exist. If the *p*-value is larger than 0.05, we cannot conclude that a significant difference exists. Data analysis was conducted using Microsoft Excel for iMac, Version 16.30.

### Inclusion and Ethical Considerations

The inclusion criteria were all cases with a component of Non-Small Cell Lung cancer and/or adenocarcinomatous differentiation or those in which a pulmonary carcinoma or adenocarcinomatous component could not be excluded, verified by a pathologist before being included in the study. Approval from the Institutional Ethical Committee was obtained prior to data collection during data collection from all three institutions, no other clinicopathologic data were collected for this analysis except those necessary for this study.

## Findings Analysis

**Figure 1:**
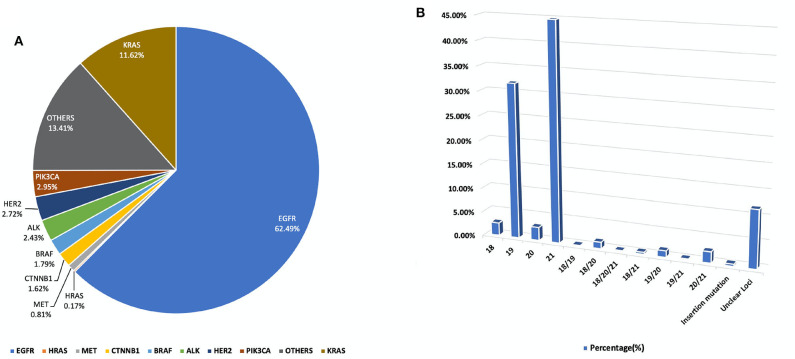
**(A)** A pie chart representing the percentage distribution of all studied genetic mutations among our study population. **(B)** Outlines the percentage duplication and triplication of EGFR mutations among patients in our cohort on Exons 18, 19, 20, and 2. For a detailed breakdown of mutation counts.

**Figure 2:**
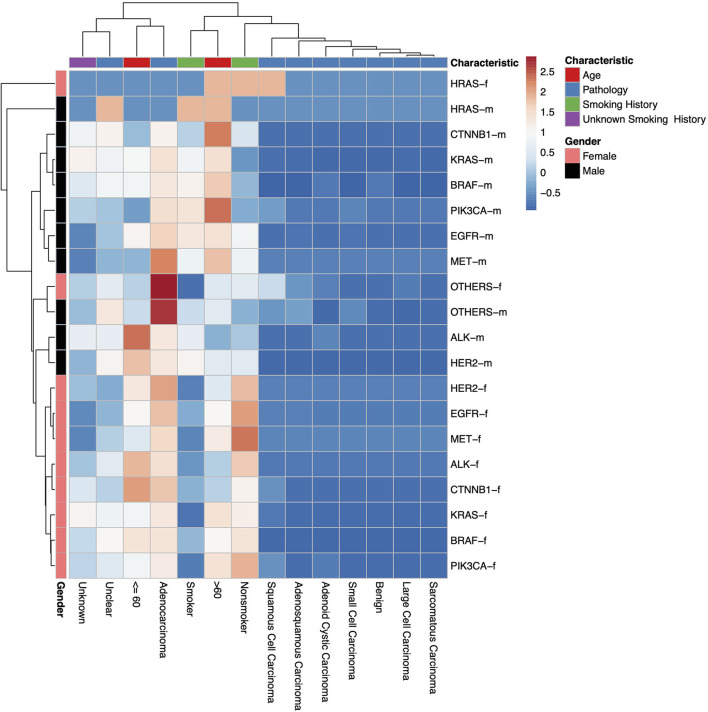
A heatmap dendrogram showing the density of distribution between all observed genetic mutations, gender, age, and pathological types observed in our study population. “-m” = males, “-f” = females. “Unknown” indicates where there is no recorded pathological type, “Unclear” indicates where the recorded pathological type was unclear, uncertain or not sufficiently identified.

In our study, all three hospitals had data on the following genetic mutations EGFR, ALK, KRAS, HER2, PIK3CA, MET, BRAF, HRAS, CTNNB1. However, data about other mutations were collected from one individual hospital. It was observed that the preponderance of genetic modifications was not evenly distributed. EGFR, KRAS, ALK, BRAF, HER2, PIK3CA, related mutations occurred in higher frequency, percentage population of patients can be found in Figure 1 and, gene localization can be found in Figure 2.

### Detected Genetic Mutation Loci

**Figure 3:**
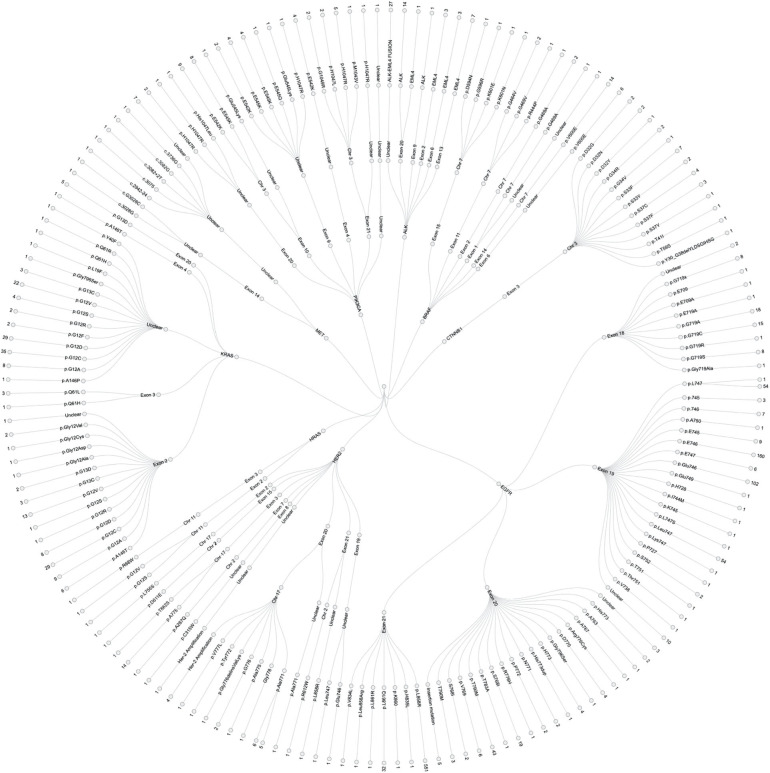
Detailed infographic, showing all genetic mutations observed during our study of major findings EGFR, ALK, KRAS, HER-2, PIK3CA, BRAF, BRAF, CTNNB1, HRAS, and MET mutations in our sample population. This illustrates specific affected genes, chromosomal positions, Exons and number of cases per location. See Figure 1 for percentage population of each genetic mutation.

In this study, a total of 1,729 test reports were analyzed, including 1,081 (62.49%) EGFR mutation carriers. **EGFR mutations** were classified in relation to the locations of point mutations. Exon 18, Exon 19, Exon 20 and Exon 21 mutations were found in 32 (2.61%), 390 (31.86%), 34 (2.78%), and 548 (44.77%) cases, respectively. However, there were cases of multiplicity where EGFR activity was observed in multiple exons such as Exon 18 and 19, 18 and 20, 18, 20 and 21, 18 and 21, 19 and 20, 19 and 21, 20 and 21. Details can be found in Figure 1B. Also, in our cohort, 11.62% of patients were found to bear **KRAS mutations** and 32.8% of them were found to exhibit mutations at the p.G12C position. It was noted that the least occurring mutations included p.Y40F, p.Q61R, p.Q61L, p.Q61H, p.L19F as illustrated in Figure 2. ALK mutations were detected as follows: 42 (2.43%) EML4-ALK. Gene localization revealed *n* = 27 as fusion genes, while others were identified on Exons 20 (*n* = 15), Exon 13 (*n* = 7), Exon 2 (*n* = 3), Exon 6 (*n* = 3), and Exon 9 (*n* = 1). The **BRAF gene** was observed to comprise 1.79% (*n* = 31) of lung cancer-associated gene mutations in our cohort and 64.52% of mutation cases were male and mainly comprised of p.V600E at Exon 15. Furthermore, non-p.V600E mutations (*n* = 11, 34.37%) occurred between Exon 15 and Exon 11 as seen in Figure 3. **PIK3CA** prevalence in our sample population was 2.95% just above the range of 1.5–2.6% (9) and Exon 9 prevalence was previously reported as 78.6% (9) contrary to our findings of 33.33% patients affected at Exon 9, in our study population. Exon 10 affectation was most observed, recorded at 35.29%. Gene localizations are shown in Figure 3 with p.E542K and p.E545K accounting for the majority of occurrences. Among patients with **MET anomalies**, *n* = 7 (64.3%) were *MET*amp, other MET modifications recorded equal distribution among our patients as follows; *n* = 1 MET c.3736G>C p.D1246H, an amino acid missense substitution mutation on position 1,246, *n* = 1 c.3082G>C p.D1028H, an amino acid substitution mutation at position 1,028, *n* = 1 c.2942-24_2942-1del24, *n* = 1 c.3082+2T>C, an intronic coding sequence mutation and *n* = 1 c.3075_3082+4del12, *n* = 1 c.3028G and *n* = 1 c.G3028C. Forty-seven (*n* = 47) **HER 2 mutations** were detected. Among the most significant mutations are: *n* = 4 (8.51%) HER2 gene amplification, *n* = 14 (29.79%) p.A775_G776insYVMA in-frame insertion mutations, *n* = 10 (21.28%) p.Ala771_Met774dup. HRAS Mutations were seen at positions p.G12S, p.G12V, and p.R68W with an equal frequency of *n* = 1 (33.3%) each.

### Gender Associations With Detected Mutations

Gender associations among all examined patients revealed *n* = 771 (44.62%) males and *n* = 957 (55.38%) females, as seen in Table 1.

**Table 1:** Illustrates the correlation between the individual genetic mutations, the gender distribution of each mutant gene, smoking history among or lack thereof among patients, and the age distribution.

		**Gender (*****n*** **=** **1,728)**					**Smoking history (*****n*** **=** **1,501)**					**Age (*****n*** **=** **1,725)**				
**GENE**	***n* (%)**	**Female (%)**	**Male (%)**	n^*^	***p*-value**	***u***	**Non-smoker (%)**	**Smoker (%)**	n^**^	***p*-value**	u*	**** < =** 60(%)**	**>60(%)**	n^***^	***p*-value**	u*
EGFR	1,081 (62.49%)	685 (63.43%)	395 (36.57%)	1,080	<0.0001	1	774 (74.71%)	262 (25.29%)	1,036	<0.0001	45 (4.16%)	526 (48.79%)	552 (51.21%)	1,078	0.0207	3
ALK	42 (2.43%)	24 (57.14%)	18 (42.86%)	42	0		21 (70.00%)	9 (30.00%)	30	0.0004	12 (28.57%)	33 (78.57%)	9 (21.43%)	42	0.0854	
KRAS	201 (11.62%)	58 (28.86%)	143 (71.14%)	201	<0.0001		44 (42.31%)	60 (57.69%0)	104	<0.0001	97 (48.26%)	88 (43.78%)	113 (56.22%)	201	0.7718	
HER2	47 (2.72%)	30 (63.83%)	17 (36.17%)	47	<0.0001		30 (78.95%)	8 (21.05%)	38	<0.0001	9 (19.15%)	28 (62.22%)	17 (37.78%)	45	0.9234	
MET	14 (0.81%)	8 (57.14%)	6 (42.86%)	14	<0.0001		11 (78.57%)	3 (21.43%)	14	0.0001	0 (0.00%)	4 (28.57%)	10 (71.43%)	14	0.4071	
HRAS	3 (0.17%)	1 (33.33%)	2 (66.67%)	3	0.4226		1 (33.33%)	2 (66.67%)	3	0.4226	0 (0.00%)	0 (0.00%)	3 (100.00%)	3	–	
BRAF	31 (1.79%)	11 (35.48%)	20 (64.52%)	31	<0.0001		9 (42.86%)	12 (57.14%)	21	0.0031	10 (31.25%)	14 (43.75%)	18 (56.25%)	32	0.7108	
PIK3CA	51 (2.95%)	23 (45.10%)	28 (54.90%)	51	0		20 (52.63%)	18 (47.37%)	38	<0.0001	13 (25.49%)	15 (29.41%)	36 (70.59%)	51	1	
CTNNB1	28 (1.62%)	12 (42.86%)	16 (57.14%)	28	<0.0001		11 (64.71%)	6 (35.29%)	17	0.0206	11 (39.26%)	14 (50.00%)	14 (50.00%)	28	0.4983	
OTHERS	232 (13.41%)	105 (45.45%)	126 (54.55%)	231	<0.0001	1	87 (43.50%)	113 (56.50%)	200	<0.0001	31 (13.36%)	109 (46.72%)	122 (52.4%)	229	0.2631	2
Grand Total	1,730 (100%)	957 (55.38%)	771 (44.62%)			2	1,008 (67.15%)	493 (32.82%)			228 (13.11%)	831 (48.20%)	894 (51.8%)			5

*All total percentages were calculated from patients with available data such as gender, smoking history and age. n–Total number of patients per gene group within our study population*.

n*- *Total number of males and females per gene group whose gender were recorded*.

n**- *Total number of smokers and non-smokers per gene group with recorded smoking history*.

n***- Total number of under 60 and over 60 patients per gene group with recorded age. u*-Unknown or unclear data at the time of this study. u (%) The percentage shown are the percentages of the total number of studied patients n* per gene group who have no smoking history data. p-value; an alpha of 0.05 is used as the cutoff for significance. If the p < 0.05, we reject the null hypothesis that there's no difference between the means and conclude that a significant difference does exist. If the p-value is larger than 0.05, we cannot conclude that a significant difference exists.

**Figure 4:**
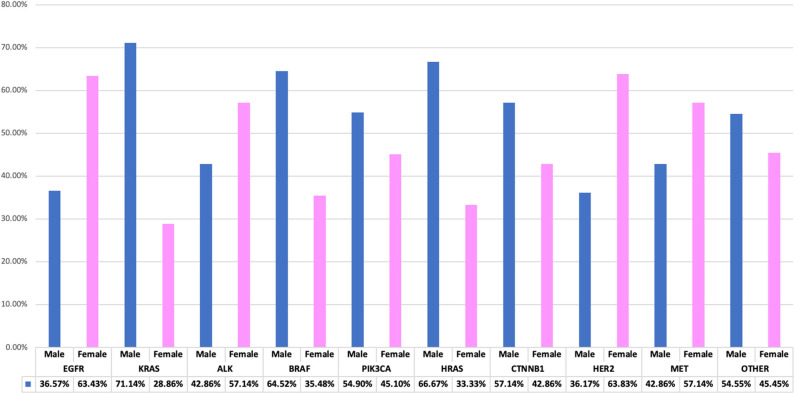
Interrelationship of observed genetic mutations with patients' gender. A bar chart illustrating genes-gender comparison, showing percentage variations between genders.

Genetic anomalies with predominant female involvement include EGFR females *n* = 685 (63.43%), ALK *n* = 24 (57.14%), HER2 *n* = 30 (63.83%), MET *n* = 8 (57.14%) while genetic activity found to involve more males are as follows: KRAS *n* = 143 (71.14%), BRAF *n* = 20 (64.52%), PIK3CA *n* = 28 (54.90%), HRAS *n* = 2 (66.67), CTNNB1 *n* = 16 (57.14%) and other genes as shown in Figure 4.

### Smoking History Interdependence With Mutations

**Figure 5:**
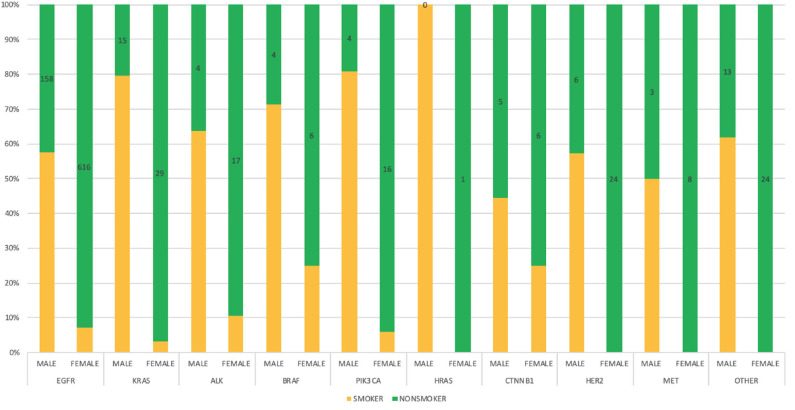
Bar chart depicting the interrelationship between genetic mutations, gender and smoking status among the study population with defined frequencies, and percentages.

Smoking histories of our cohort were also elucidated. It revealed that within our study population, there were more non-smokers (67.15%) than smokers (32.82%). However, among the male population with known smoking histories, *n* = 381 (49.35%) were smokers and *n* = 254 (32.90%) were non-smokers, while among the female population, *n* = 111 (11.60%) were known smokers and *n* = 755 (78.90%) were non-smokers. There were other patients whose smoking histories could not be ascertained at the time of this study *n* = 228 (13.11%). In our study, genetic mutations including EGFR, KRAS, ALK, BRAF, PIK3CA, HRAS, CTNNB1, HER2, and MET were predominant among male smokers, while on the other hand female non-smokers were shown to be more susceptible to all studied lung cancer-associated gene mutations. Details of gender correlation with smoking histories can be found in Figure 5.

### Age Correlation With Mutation

**Figure 6:**
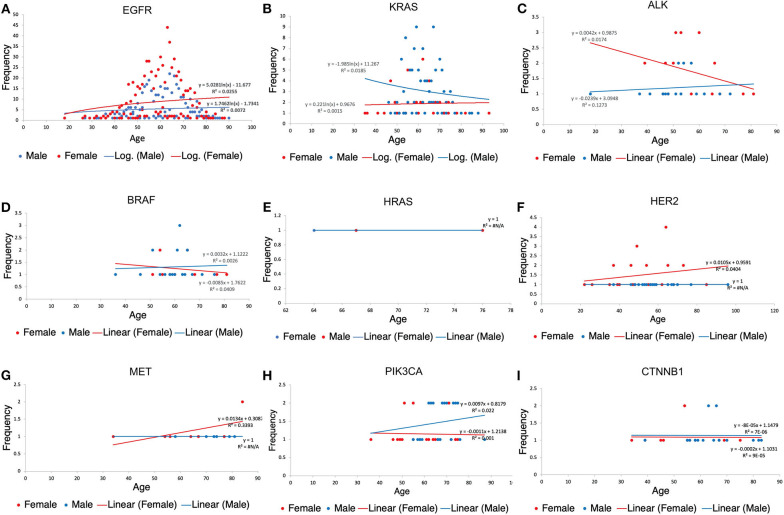
Patients' scatter plot with regression analysis showing the relationship between each gene variation, gender and age of patients. R^2^(R-squared) is a goodness-of-fit (Y = a + bX) measure for linear regression models. This statistic indicates the percentage of the variance in the dependent variable that the independent variables explain collectively. The trendline regression equation is also shown in each plot for reference. **(A)** Age distribution among EGFR patients. **(B)** Age distribution among KRAS. **(C)** Age distribution among KRAS patients. **(D)** Age distribution among BRAF patients. **(E)** Age distribution among HRAS patients. **(F)** Age distribution among HER2 patients. **(G)** Age distribution among MET patients. **(H)** Age distribution among PIK3CA patients. **(I)** Age distribution among CTNNB1 patients.

Age distribution among our study patients showed under-60 patients (48.20%) and over-60 patients (51.80%). Furthermore, among the population <60 years of age, more females (57.91%) were found to have a mutation, the same for females above 60 years of age (53.25%). Details showing the correlation between specific gene mutations, age, and patient's gender can be found in Figure 6.

### Pathological Presentation of Mutation

It was observed that majority of patients presented with LUAD lesions *n* = 1231 (71.11%), SQCC *n* = 29 (1.68%), ASC *n* = 13 (0.75%), LCC *n* = 10 (0.58%), SCC *n* = 6 (0.35%), ACC *n* = 3 (0.17%), sarcomatous carcinoma *n* = 1 (0.06%) and there were *n* = 436(25.19%) patients whose pathological types could not be ascertained at the time of this study.

LUAD lesions were most rampant among our cohort, where EGFR *n* = 847 (78.83%), ALK *n* = 25 (59.52%), KRAS *n* = 110 (54.73%), HER2 *n* = 35 (74.47%), MET *n* = 12 (85.71%), BRAF *n* = 17 (53.13%), PIK3CA *n* = 30 (58.82%), and CTNNB1 *n* = 16 (57.14%) all had predominantly adenocarcinomatous presentations while a single patient harboring HRAS gene mutation presented with SQCC. Details in Table 2. LUADs, SQCCs, LCCs, and ACCs were found to be more common among females while ASCs, LCCs, SCCs, and SCs were predominant among males. Details in Table 3. On-the-other-hand, LUAD, SQCC, and LCC occurred frequently among non-smokers, while ASC, SCC, ACC, and SCs occurred mostly among smokers as seen in Table 3.

**Table 2:** Illustrates the correlation between the individual genetic mutations, and various pathological types observed in our study population.

**GENE**	**Adenocarcinoma (%)**	**Unclear (%)**	**Squamous cell carcinoma (%)**	**Adenosquamous carcinoma (%)**	**Large Cell carcinoma (%)**	**Small Cell carcinoma (%)**	**Adenoid cystic carcinoma (%)**	**Sarcomatous carcinoma (%)**	**Grand total**
EGFR	847 (78.35%)	217 (20.07%)	5 (0.46%)	9 (0.83%)	0 (0.00%)	3 (0.28%)	0 (0.00%)	0 (0.00%)	1,081
ALK	25 (59.52%)	16 (38.10%)	0 (0.00%)	0 (0.00%)	0 (0.00%)	0 (0.00%)	1 (2.38%)	0 (0.00%)	42
KRAS	110 (54.73%)	83 (41.29%)	5 (2.49%)	1 (0.50%)	2 (1.00%)	0 (0.00%)	0 (0.00%)	0 (0.00%)	201
HER2	35 (74.47%)	12 (25.53%)	0 (0.00%)	0 (0.00%)	0 (0.00%)	0 (0.00%)	0 (0.00%)	0 (0.00%)	47
MET	12 (85.71%)	2 (14.29%)	0 (0.00%)	0 (0.00%)	0 (0.00%)	0 (0.00%)	0 (0.00%)	0 (0.00%)	14
HRAS	0 (0.00%)	2 (66.67%)	1 (33.33%)	0 (0.00%)	0 (0.00%)	0 (0.00%)	0 (0.00%)	0 (0.00%)	3
BRAF	17 (53.13%)	14 (43.75%)	0 (0.00%)	0 (0.00%)	0 (0.00%)	0 (0.00%)	0 (0.00%)	0 (0.00%)	31
PIK3CA	30 (58.82%)	14 (27.45%)	5 (9.80%)	0 (0.00%)	0 (0.00%)	1 (1.96%)	1 (1.96%)	0 (0.00%)	51
CTNNB1	16 (57.14%)	11 (39.29%)	1 (3.57%)	0 (0.00%)	0 (0.00%)	0 (0.00%)	0 (0.00%)	0 (0.00%)	28
OTHERS	139 (59.91%)	65 (28.02%)	12 (5.17%)	3 (1.29%)	8 (3.45%)	2 (0.86%)	1 (0.43%)	1 (0.43%)	232
TOTAL	1,231 (71.11%)	436 (25.19%)	29 (1.68%)	13 (0.75%)	10 (0.58%)	6 (0.35%)	3 (0.17%)	1 (0.06%)	1,728

**Table 3:** Illustrates the distribution of the pathologic presentations between gender and smoking histories.

**%**	***f* (gender)**	**Gender**	**Pathologic type**	**Smoking history**	***f* (smoking)**	**%**
59.46%	732	Female	Adenocarcinoma	Non-smoker	780	63.36%
40.45%	498	Male		Smoker	361	29.33%
	1	Unknown		Unknown	90	7.31%
51.72%	15	Female	Squamous cell carcinoma	Non-smoker	14	48.28%
48.28%	14	Male		Smoker	7	24.14%
	0	Unknown		Unknown	8	27.59%
38.46%	5	Female	Adenosquamous carcinoma	Non-smoker	4	30.77%
61.54%	8	Male		Smoker	8	61.54%
	0	Unknown		Unknown	1	7.69%
10.00%	1	Female	Large cell carcinoma	Non-smoker	6	60.00%
90.00%	9	Male		Smoker	2	20.00%
	0	Unknown		Unknown	2	20.00%
16.67%	1	Female	Small cell carcinoma	Non-smoker	2	33.33%
83.33%	5	Male		Smoker	4	66.67%
		Unknown		Unknown	0	
66.67%	2	Female	Adenoid cystic carcinoma	Non-smoker	0	
33.33%	1	Male		Smoker	3	100.00%
	0	Unknown		Unknown	0	
	0	Female	Sarcomatoid carcinoma	Non-smoker	0	
100%	1	Male		Smoker	1	100.00%
	0	Unknown		Unknown	0	
46.10%	201	Female	Unclear	Smoker	111	25.46%
53.90%	234	Male		Non-smoker	197	45.18%
	1	Unknown		Unknown	128	29.36%
	1,729				1,729	

## Discussion

The establishment of solid evidence of genetic predisposition to the risk of lung cancer has a potential clinical utility for not only stratification of the population, but also primary prevention. Our objective in this study was to elucidate, comprehend and interpret the association of various genetic mutations using an extensive exploration of organized metadata concerning gender and smoking habits and age with information gathered from three Chinese hospitals.

### Distribution of Mutations

According to Greulich H. et al. in the United States, somatic alterations of 5 lung adenocarcinoma oncogenes, KRAS, EGFR, ALK, ERBB2(HER2), and BRAF, are interestingly mutually exclusive and are represented in over 50% of lung adenocarcinomas (10) (Figure 1). Other studies also elaborate on the dominance of EGFR mutations among NSCLC patients (11, 12). On the other hand, in China, the same group of 5 oncogenes amounted to 81% of lung cancers, which is largely attributable to the high frequency of EGFR mutations including multiple occurrences of the EGFR mutations as seen in China (Table 4), vs. to non-Asian populations (13, 14).

**Table 4:** Outlines the multiplicity of distribution of EGFR mutations among patients in our cohort on Exons 18, 19, 20, and 21.

**EGFR Exon**	**Mutation Counts**	**Percentage (%)**
18	32	2.61%
19	390	31.86%
20	34	2.78%
21	548	44.77%
18/19	1	0.08%
18/20	16	1.31%
18/20/21	2	0.16%
18/21	5	0.41%
19/20	16	1.31%
19/21	3	0.25%
20/21	28	2.29%
Insertion mutation	5	0.41%
Unclear Loci^*^	144	11.76%
Total Count^**^	1,224	

* *Unclear loci indicates patients whose gene analysis detected an EGFR mutation but which specific Exonal position remains unclear*.

** *Total count refers to the total count of occurrences of mutations including the duplicates and triplicates among 1,081 recorded EGFR patients*.

Also according to the AACR project GENIE consortium database, EGFR is mutated in 22.17%, ALK is mutated in 5.05%, BRAF is mutated in 5.34%, ERBB2 (HER2) is mutated in 4.12%, HRAS is mutated in 0.43%, KRAS is mutated in 29.7%, MET is mutated in 5.18%, NRAS is mutated in 1.14% and PIK3CA is mutated in 7.47% of non-small cell lung carcinoma patients. In comparison to our results seen in Figure 1, there is a considerable departure, with one of the obvious causes being ethnic variations in genetic makeup (15).

The results of this study reflected the tremendous data available for the study of mutations in China. This is mostly due to the widespread availability of testing centers at various hospitals across the country which has resulted in early detection of genetic mutation associated with non-small cell lung cancer in the affected population.

Our study further confirmed that Asians who harbor NSCLC have similar genetic components (16–19), as also demonstrated in a study conducted among Korean patients (14). In France, 66% of V600E mutations were observed among BRAF mutated patients (20). This is important because V600E is an oncogenic mutation and a major target of specific inhibitors. Little is known about the clinical significance of BRAF Non-V600E mutations' role in lung cancer, however, it's recently been associated with colorectal cancer (21).

### Smoking History

Cigarette smoking—is by far the leading cause of lung cancer, accounting for about 80 to 90% of lung cancer cases in the United States and other countries where cigarette smoking is common (22).

There is a known association between the 15q24 susceptibility locus and lung cancer. However, it is unclear whether it is direct (i.e., there is a gene in that region that causes lung cancer) or indirect (i.e., there is a gene in that region that causes tobacco addiction, which in turn causes lung cancer) remains to be determined (23).

Generally, it was observed in our study that more female non-smokers were more at risk of lung cancer compared to men. There is no clear reason for this perhaps secondhand smoking may have played a significant role. This is in sharp contrast to a previous study in the United Kingdom where it was reported that Moderate and heavy smoking carries a higher risk of lung cancer in women than in men (24).

However, multiple previous studies have shown a higher incidence of EGFR mutations in female non-smokers of Asian origin (9, 25, 26).

Tumors that contain the EML4-ALK fusion oncogene or its variants are associated with specific clinical features, including never or light tobacco smoking (16, 27).

In a previous report (27), a significantly higher rate (22%) of ALK rearrangements in never or light smokers with NSCLC, suggesting a strong association between ALK rearrangements and a never or light smoking history. However, little is known about associations between non or never smokers and ALK mutations (19, 28). On the other hand, another study of 7/208 patients in china showed smokers were more likely to present with EML4-ALK mutations (12). This was a sharp contrast however its worth mention that the said study population was quite small. Our finding showing *n* = 755 (92%) female non-smokers vs. *n* = 60 (8%) female smokers affirms this theory as shown in Table 3.

Notably, KRAS mutations were found predominantly in male smokers and female non-smokers. This could be due to the fact that pulmonary carcinomas from never-smokers are more likely to be transition mutations, unlike those in lung cancers from smokers, which commonly are transversion mutations (4, 29).

In Japan, as shown in a study conducted on BRAF gene mutations conducted on NSCLC patients, 0.8% of the population had mutations and the majority of the patients were male smokers (14). However, in our study, 1.79% of patients were found to be BRAF positive and most were also male smokers.

In contrast to previous enumerated genetic aberrations, we have seen that MET genetic activity in our cohort falls slightly short of the expected range; 0.81% in our study. MET mutations have recently been shown to occur in 3 to 4% of NSCLC adenocarcinomas, 2% of squamous cell carcinomas, and 1 to 8% of other subtypes of lung cancers (27). Noteworthy, it's more common among non-smokers (30), indicating 55.7% MET mutations among non-smokers vs. 61.11% non-smokers who were found to harbor MET mutations in our cohort.

HER2 mutations were observed in 2.72% of NSCLCs, particularly in younger patients, and those with no history of smoking which is within the 2–4% range seen in Japan (6, 31). Elsewhere in China, it was also found that 1.9% of NSCLC patients had HER2 activity among never smokers who happen to be no more than 60 years old (32).

Despite the rarity of the CTNNB1 mutations, we were able to find its occurrence in 1.62% of our study population which is quite similar to 1.5% obtained in Germany (10). It is noteworthy that genetic alteration of the β-catenin gene (CTNNB1) in human lung cancer was first elaborately reported when four alterations were found in Exon 3 (7). In our study population, all 28 patients had mutations on Exon 3 which is the target region of mutation for stabilizing β-catenin. More non-smokers 39.2% bore CTNNB1 mutations compared to smokers 21.42%. However, for more accurate study, it's important to clearly isolate never smokers from previous smokers, light smokers, and current smokers in order to have unclouded scrutiny of patients' interconnection with genetic mutability.

### Gender Interrelationship

A study of 50-year trends in smoking-related mortality in the USA found that males had higher relative risks of smoking-related lung cancer mortality were higher compared to females (33). In contrast, a recent study in Korea suggested that gender differences in the impact of smoking on lung cancer risk exist and differ by histological subtype (34). Analyses of a large primary care database in the UK showed that moderate and heavy smoking more strongly increases the risks of lung cancer in women than in men (35).

It was previously reported that subject to availability of data in regions were studies were carried out, EGFR mutation frequency in patients with NSCLC/ LUAD was higher in women compared with men: Europe, 22 vs. 9%; Asia-Pacific 60 vs. 37%; Indian subcontinent, 31 vs. 23%; Africa, 48 vs. 8%; and North America 28 vs. 19% (36). In our study, EGFR patients also demonstrated a larger female to male ratio where we have 63.37% females vs. 36.54% males. Among our KRAS affected study population, we found that more males were associated with KRAS mutations to the tune of 71.14% male and 28.86% females which corresponds to an earlier finding among Turkish patients where (58%) male and (42%) female were identified among KRAS patients (15). RAS oncogene has three known isoforms as Harvey- RAS (HRAS), Kirsten–RAS (KRAS) and NeuroblastomaRAS (NRAS). HRAS mutations are observed very rarely in lung cancers (<1%) (37). As seen in our cohort, only 0.1% of patients had HRAS mutation, both of whom were male smokers. Little is known about this gene and its lung cancer affectation.

### Correlation With Aging

Cancer is a disease associated with aging—the majority of cancer diagnoses and deaths occur in people older than 65 years (38). Of particular interest is the finding that Asian women with the EGFR mutation developed adenocarcinoma at an earlier age than other lung cancer patients (5, 39). In our study, EGFR anomalies were detected in young patients ranging from 18 to 87 years, even though the median age was about 60 years (2). HER2 alterations were also spotted among younger patients with age ranging from 22 to 96 years. Of note is an 18-year-old male patient who was the youngest patient with ALK mutation in our study group. Numerous explanations have been offered as to the biologic connection between cancer and aging, including extended exposure to carcinogens (13), increased susceptibility to oxidative stress (40), immune dysregulation (41). While these explanations for the link between cancer and aging are plausible, they do not pinpoint the reason why one older adult is more susceptible to cancer than another. Furthermore, the association between cancer and aging is complex. It however appears that age independently associates with EGFR mutation among lung cancer (42).

### Pathological Presentations

**Figure 7:**
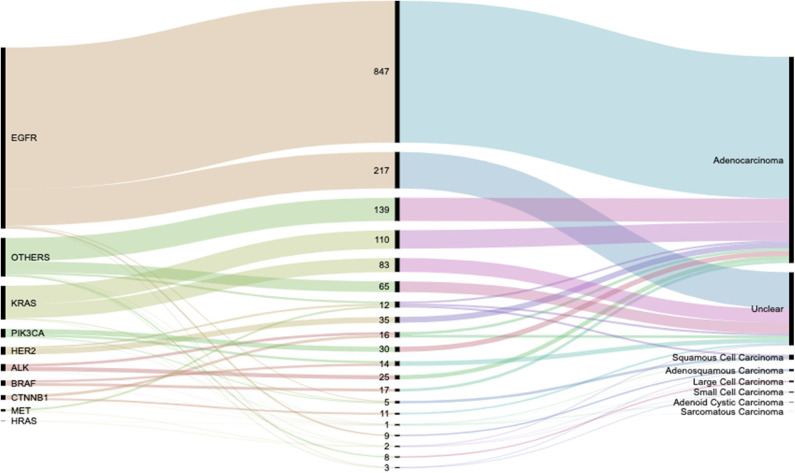
Alluvial diagram showing the flow distribution of genetic activity between the various observed pathological types. Detailed weights and percentages can be found in Table 2.

Lung cancers are traditionally divided into non–small cell carcinoma (NSCC) and SCC (small cell lung carcinoma, SCLC), with the former accounting for 80% of the cases and the latter accounting for the remaining 20%. Lung cancer can be diagnosed pathologically either by a histologic or cytologic approach (43), of which in our study the histologic approach was used. There exists strong disparities between lung LUAD in the Europeans vs. East Asians which could mainly be due to the disparity in smoking habits between both populations with the majority of the driver genes being EGFR and KRAS (20). In a Korean study using data from the Korea Central Cancer Registry (2), it was reported that a higher risk for having ever smoked was observed for squamous-cell and small-cell carcinoma in both men and women. However, in that study no mention was made about ASCs, on the contrary, in our study, we found that for SQCC patients, non-smokers were more at risk. Worthy of mention is the high frequency of LUADs, SQCCs, and LCCs among non-smokers In China, we were able to outline eight pathological types during our study with LUADs proving to be the most prevalent pathological presentation in our cohort, Figure 7. There was also notable risk association between smoking and incidences of ASCs and SCCs. These and the gender discriminations of lung cancer pathologies will be subject of further study.

## Conclusion

Lung cancer-associated genetic mutations are widespread in China. Detection is facilitated by the availability of screening centers in various hospitals including the hospitals where our study population was sampled. EGFR is one of the most prevalent genetic alterations among lung cancer patients, even though other genetic aberrations also exist. EGFR, ALK, HER2, and MET anomalies were more prevalent among females while KRAS, HRAS, BRAF, PIK3CA, CTNNB1, and other genes were more prevalent in males. Genetic mutations such as EGFR, KRAS, ALK, PIK3CA, HRAS, HER2, CTNNB1, BRAF, MET are more common in female non-smokers, with some mutations existing in non-smoking patients. ALK, KRAS, and BRAF genes anomalies were predominantly found among patients younger than 60 years, while the other genes in our study were predominant among older patients or showed no significant age bias. Subsequent to this expose detailing the peculiarities of Chinese patients' genetic affiliations to lung cancer, more work needs to be done in collecting more detailed smoking histories to further increase the accuracy for future work.

## Data Availability Statement

The datasets generated for this study are available on request to the corresponding author.

## Ethics Statement

The studies involving human participants were reviewed and approved by Scientific Research Committee of Beijing Shijitan Hospital. The patients/participants provided their written informed consent to participate in this study.

## Author Contributions

IA: writing—original draft, writing—review and editing, and visualization. XX: conceptualization, methodology, and supervision. JF, LH, JG, ZD, and JH: data curation and formal analysis. HD, TJ, and JW: project administration. XZ and MM: formal analysis and validation. XM: funding acquisition. HW, LP, and DL: resources. RG, ZX, DZ, CQ, and YM: data curation and formal analysis. HM: supervision. All authors contributed to the article and approved the submitted version.

## Conflict of Interest

The authors declare that the research was conducted in the absence of any commercial or financial relationships that could be construed as a potential conflict of interest.

## Abbreviations

LUAD: Lung Adnocarcinoma
ACC: Adenoid cystic carcinoma
ASC: Adenosquamous Carcinoma
LCC: Large Cell Carcinoma
SCC: Small Cell Carcinoma
SC: Sarcomatoid Carcinoma
SQCC: Squamous Cell Carcinoma.
